# Assessment of the population of *Ostrea edulis* in Sweden: A marginal population of significance?

**DOI:** 10.1002/ece3.5824

**Published:** 2019-11-19

**Authors:** Linnea Thorngren, Per Bergström, Thomas Dunér Holthuis, Mats Lindegarth

**Affiliations:** ^1^ Department of Marine Sciences University of Gothenburg Gothenburg Sweden; ^2^ Department of Marine Sciences Tjärnö University of Gothenburg Strömstad Sweden

**Keywords:** benthic habitat, conservation, *Ostrea edulis*, sampling methods, sustainable management, towed video

## Abstract

1. The European flat oyster *Ostrea edulis* is an economically and ecologically important species subjected to extensive protection and restoration efforts, due to sharp population declines in Europe. In Sweden, *O. edulis* occurs at the northern fringe of its range. Knowledge of the distribution and abundance of the species is limited, and the size of the population has never been estimated. Oyster fishery sustainability has never been assessed.
2. Using a random sampling approach and towed video, we collected data on oyster occurrence at 435 sites to estimate abundance and distribution of *O. edulis* in the Swedish Skagerrak region. Furthermore, the size of the population was assessed and the current management and legislation strategy of the species was analyzed.
3. Living *O. edulis* was found in 27% of all sampled sites above 6 m, and the size of the population was estimated to 36.6 ± 16.3 million individuals (total population ± *SE*). The distribution was patchy, and approximately 60% of the population was found in oyster bed densities (≥5 oysters/m^2^), which corresponds to around 1% of the sampled sites.
4. The nondestructive sampling method and representative design provided useful estimates of population size and error, which indicate that the marginal population of *O. edulis* in Sweden constitutes a significant part of the remaining European population. We argue that the relatively good status of the Swedish population can be explained by (a) private ownership of fishing rights, (b) a small‐scale fishery that exploits <0.5% of the estimated population annually, conducted using nondestructive methods, and (c) parasite‐free waters, potentially due to effective prevention of spread of infection.

**Open Research Badges:**

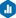

This article has earned an Open Data Badge for making publicly available the digitally‐shareable data necessary to reproduce the reported results. The data is available at https://osf.io/jgpxw/?view_only=d070b45802a4426da028efffde3d0f76.

## Graphical abstract

Using a nondestructive sampling method and representative design, the Swedish population of the European flat oysters, *Ostrea edulis*, was assessed. The study indicates that the status of the population is relatively good, compared to most populations in Europe. We argue that (a) private ownership of fishing rights, (b) a small‐scale, nondestructive fishery, and (c) parasite‐free waters contribute strongly to this finding.

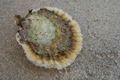

## 1 INTRODUCTION

In coastal areas around the globe, filter‐feeding bivalves often constitute important components of soft and hard substratum, in both inter‐ and subtidal benthic communities (Gosling, 2015). Although the species differ among biogeographic regions, their contributions to supporting and regulating ecosystem services, for example, benthic–pelagic coupling through filter‐feeding and maintenance of water quality (Newell, 2004), the cycling of nutrients (Smaal & Prins, 1993; Jansen et al., 2019), prevention of erosion (Meyer, Townsend, & Thayer, 1997), and provision of habitat for invertebrate and fish species, are of great importance (Coen, Luckenbach, & Breitburg, 1999; Peterson, Grabowski, & Powers, 2003; Stunz, Minello, & Rozas, 2010).

The European flat oyster, *Ostrea edulis* (Linnaeus 1758, Figure 1), native to Europe, occurs naturally from the Norwegian Sea in the north to Morocco in the south, and east through the Mediterranean Sea to the Black Sea (OSPAR, 2009). The species is mainly found in sheltered sedimentary environments, in the subtidal or the lower intertidal zone, where it creates structurally complex habitats, which provides important ecosystem functions (Peterson et al., 2003) and references therein). *Ostrea edulis* also has great commercial and cultural significance, due to long‐standing capture fishery and aquaculture (FAO, 2019) and, more recently, the development of tourism linked to the species (Goolaup & Mossberg, 2017). In the last decade, capture fishery production has been estimated at 1,000–2,000 tonnes annually with the corresponding value for aquaculture production 2,000–3,000 tonnes (FAO, 2019). Due to this limited supply, the species is highly valued and therefore occupies a niche market which is still an important industry component.

**Figure 1:**
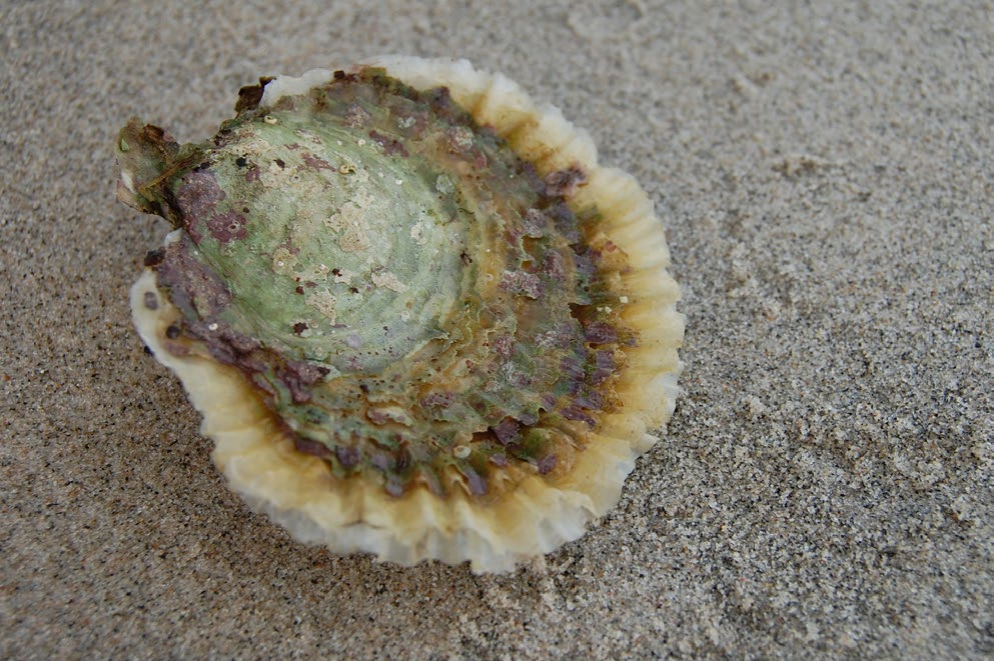
An adult individual of the European flat oyster, *Ostrea edulis*, photographed on the beach

The high value attributed to the species has led to overexploitation and contributed to the decline of European *O. edulis* populations observed during the 19th century (Airoldi & Beck, 2007; OSPAR, 2009; Beck et al., 2011). The recovery of natural populations has been slow, mainly due to insufficient larval production (i.e., lack of broodstock) and degraded habitats, which reduce the available area for larvae settlement (Eno et al., 2013; Pogoda et al., 2019). Another important factor was the introduction of protozoan parasites such as *Bonamia ostreae*, which during the 1970s severely reduced wild populations of *O. edulis* (Edwards, 1997; Smaal, Kamermans, Have, Engelsma, & Sas, 2015). Where present, *B. ostreae* is still believed to be the most important obstacle to stock recovery (Laing, 2005). In northern Europe, the only remaining well‐documented populations which still exist occur in a number of sea loughs and estuaries within the British Isles (Kennedy & Roberts, 1999; UMBS, 2007; Smyth, Roberts, & Browne, 2009; Tully & Clarke, 2012), in the Limfjord in Denmark (Kristensen & Hoffmann, 2006) and in Norway (Bodvin, Mortensen, Jelmert, Strand, & Grefsrud, 2011).

Based on its economic and ecological importance, its history of exploitation, the increasing pressures due to human activities, and general degradation of coastal benthic habitats, populations, and beds of *O. edulis* are identified as a priority species/habitat by the OSPAR Convention since 2003 (OSPAR, 2009). Oyster beds also receive protection through the EU Habitats Directive (92/43/EEC 1992); “Biogenic reefs” as well as through national legislation, for example, the UK Biodiversity Plan (JNCC, 2014). In addition, several restoration projects are currently being carried out in Europe, for example, the DEEP project in Scotland, the FOREVER project in France, the ENORI and Solent Oyster projects in England, and several projects in the Netherlands (NORA, 2019).

In Sweden, the *O. edulis* population can be considered to be on the margin of the species’ distributional range due to its northeastern location and its vicinity to the brackish Baltic Sea (Johannesson, Rödström, & Aase, 1989; Rödström & Jonsson, 2000). The species is only found in the northern part of the Swedish west coast, where the salinity is high enough and numerous small islands contribute to a patchy distribution pattern (Lindegarth, Dunér Holthuis, Thorngren, Bergström, & Lindegarth, 2014). Historical and contemporary knowledge about the geographical and vertical distribution and abundance of *O. edulis* in the region is inadequate, and the understanding of fundamental life‐history characteristics such as recruitment is largely anecdotal (but see Rödström & Jonsson, 2000). Hence, the scientific basis for adaptive management of this population in relation to existing exploitation pressures must be considered very weak. The OSPAR and EU commitments are partly implemented in regulations of protected areas, but the most important legal instrument is the Swedish “fisheries law,” stating that fishing for oysters is reserved to the landowner (§ 9, SFS :787). This is the only species in the sea covered by private fishing rights. Additionally, there is a law on minimum catch size of 6 cm (§ 16, FIFS, 2004:36). Fishing has never been regulated by quotas but a handful of diving fishermen, usually leasing the rights from landowners, have harvested approximately 10 tonnes of *O. edulis* annually for the last decade (Swedish Agency for Marine and Water Management, 2018).

In addition to the native oyster species, the alien *Magallana (Crassostrea) gigas* appeared in great numbers on the Swedish west coast in 2006 and is now established in the region (Wrange et al., 2010; Strand & Lindegarth, 2014). This non‐native species often co‐occurs with *O. edulis* or the blue mussel *Mytilus edulis* (pers. obs.), and its interaction with the two native bivalve species in this region is under assessment. Interestingly, the Swedish legislators have chosen not to distinguish between the two species (i.e., fishing rights are reserved to the landowners).

The aim of this paper was to assess the abundance and spatial distribution of the native oyster *O. edulis* in Sweden. A stratified random sampling design and towed video were used to collect representative and quantitative estimates of *O. edulis* abundance at a large number of sites. Using these samples, we were able to estimate the population size and associated sampling error of *O. edulis* in the area. We show, among other things, that the species is frequently found but highly variable in abundance in the region, and that this marginal population may constitute a significant part of the decimated North Sea *O. edulis* population. These insights can provide the basis for the development of a sustainable exploitation and conservation strategies of this key species in a previously less studied area in its distributional range.

## 2 MATERIALS AND METHODS

### 2.1 Study area and design

**Figure 2:**
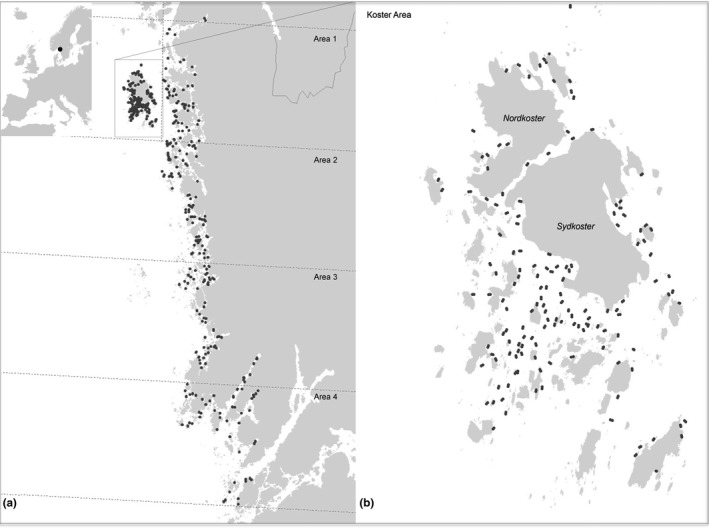
Map of the study area showing (a) regional overview of the study area, including subarea divisions and the location of all sampled sites and (b) close‐up of the densely sampled Koster area

This study was performed in the Swedish Skagerrak region, an approximately 90‐km‐long coastal strip where the main proportion of the Swedish *O. edulis* population occurs (Figure 2). The area is characterized by a small tidal range (amplitude ≈ 20 cm) and fluctuating salinities (normally 20–30 psu in the surface water). An extensive archipelago, with both rocky and sandy shores, contributes to large variations in wind and wave exposure. Mean summer temperatures in the surface water usually do not exceed 20°C, and temporary ice‐cover may occur during December–March in sheltered parts of the coastal area.

The data and analyses presented in this paper originate from two separate field inventories. The first inventory covers the whole coastal region described above, while the second is concentrated to the Koster islands. The second part was planned after the initiation of the first part as a result of an external assignment from the County Administrative Board of Västra Götaland, aimed at estimating the distribution and occurrence of oysters within the Kosterhavet National Park (Lindegarth et al., 2014). Since sampling programs were planned individually, which resulted in large differences in sampling intensity, analyses were done separately despite the use of identical field methods and video‐analyses. This was to avoid bias in overall population estimates and overemphasis on patterns observed in more intensely sampled areas. The inventory was carried out in accordance with the “Permit on scientific research and collection of red‐listed species in Kosterhavet National Park in the municipalities of Strömstad and Tanum” given by the County Administration Board of Västra Götaland (Permit Number: 521–1553–2014).

### 2.2 Sampling design

In order to achieve a representative estimate of the oyster population throughout the area, sites were selected using ArcGIS 10.0 (ESRI, 2010). According to local fishermen, high abundances of *O. edulis* are rarely found below 8 m. Sampling was therefore constrained to areas shallower than 10 m, and to areas classified as moderately exposed or less exposed according to the Swedish classification of wave and wind exposure (Wennberg et al., 2006). These restrictions incorporate all suitable habitats for this species in the study area. The samples were stratified into the depth strata 0–3, 3–6 and 6–10 m. Apart from being ecologically relevant for the distribution of this species, these strata are standard depth intervals in nautical charts. Thus, these strata, as well as their estimated areal extent, were defined and available a priori. This was important to allow estimation of population size from mean densities and areal extent. Otherwise, no restrictions were imposed on sampling based on substrate characteristics, despite the fact that it is well known that *O. edulis* is not found on hard substrata in the area. Because no reliable information exists on the distribution of different substrates in the area, this had to be estimated from the data a posteriori, and potentially rocky sites within the defined depth and exposure criteria were therefore included in the sampling program. Sampling was done during 2013 and 2014, but as a result of the sampling in the first year, when extremely few oysters were found in the deepest interval, the depth stratum 6–10 m was excluded during the second year.

### 2.3 Field methods

Sampling of oysters was carried out using a method developed and evaluated during spring 2013, described in (Thorngren, Holthuis, Lindegarth, & Lindegarth, 2017), with the difference that a flat‐bottomed boat with a step‐less electric engine was used to maneuver the sled. Once in position at a sampling site, a random number table (with the accuracy of 0.1 m) was used to determine the specific sampling depth within the strata. If this depth was found within 100 m from the original sampling position, two 20‐m video transects were recorded along the depth curve, using a sled with a downward‐facing, high‐definition (HD) GoPro Hero 2 camera (color, 1,080 p) mounted 50 cm from the seafloor, giving a picture frame size of 0.45 × 0.8 m. This gave a total filmed area of 32 m^2^ per site, which result in an estimated error of approx. 0.6 oysters/m^2^ (Thorngren et al., 2017). Speed was kept below 0.4 knots to obtain sufficient image quality. Start and end points for each video transect were determined using GPS.

The aim of the sampling design was to achieve a sampling program representative of habitats between 0.5 and 10 m. Nevertheless, for practical reasons we were not able to sample at some sites. On five occasions, filming could not be carried out because the sampling site was located in a harbor, too narrow or crowded with boats for sampling to be conducted. Such sites were excluded from the study. Additionally, there were instances when the selected site was not sufficiently large to practically allow filming of two 20‐m transects at a given depth. These were typically underwater rocks with or without a dense cover of macroscopic algae. Because rocky environments are known not to contain *O. edulis*, they are still a part of the population of sites. Therefore, transects were not filmed at these sites but *O. edulis* was recorded as absent following visual inspection.

### 2.4 Methods for extracting video data

The videos were imported to iMovie (version 9.0.9), where a number of living and dead *O. edulis* were estimated by a trained observer as described in Thorngren et al. (2017). The analysis included individuals larger than approximately 4 cm, since identifying smaller individuals has been shown difficult on video (Sallén Lennerthson & Lindegarth, unpublished data). Data on living oysters were collected in order to assess the current population of the species. Information on dead oysters was collected mainly for the purpose of identifying potential habitats or areas where it previously occurred. Additional data were also collected to assess the cover of different types of substrate and vegetation, as well as the presence of various algae and sea grass species. This was done by subsampling ten frames per transect and estimating the cover of substrate classes (mud, sand, gravel, shell hash, and boulders or rock) and vegetation to the nearest 25% in each frame.

### 2.5 Data analysis

Based on the data collected in the two studies, we estimated frequency of occurrence, abundance, and total population size of *O. edulis* at different depths and in different parts of the region. General patterns of abundance related to habitat characteristics, that is, substrate and vegetation cover, were assessed using nonmetric multidimensional scaling using the Bray–Curtis dissimilarities in the “metaMDS”‐function (“vegan” library version 2.5–4; Oksanen et al., 2013) implemented in the R software (R Core Team, 2017).

Hypotheses about differences in abundance of *O. edulis* among areas and depth strata were tested using the data from the field observation and analysis of variance (ANOVA) according to procedures described in Underwood (1997). The model involved two fixed factors: one representing geographical differences in the region, (“Area”; levels Areas 1–4 and Koster) and one representing the three strata: (“Depth”; levels 0.5–3, 3–6, and 6–10 m) and one random factor “Site” (nested within Area × Depth). The definition of four different areas was done in order to allow assessment of differences among latitudinal areas. These areas were, however, not defined prior to sampling, and therefore, the data are slightly unbalanced with respect to areas. Student–Neuman–Keuls (SNK) tests were used a posteriori to test for differences among means of significant main effects and interactions.

The representative data from the field study were used in combination with GIS information on the areal extent of depth strata within the five areas (Table 1). Because random, representative samples were allocated separately to depth strata in the two studies, mean estimates (${\bar{y}}_{\text{St}}$) and variances ($s_{\text{st}}^{2}$) were calculated separately for depth strata within each study before they were combined into overall estimates of (${\bar{y}}_{\text{total}}$) and variances $V\left({\bar{y}}_{\text{total}}\right)$ according to procedures for stratified sampling described in Cochran (1977):

(1) $${\bar{y}}_{\text{total}}=\sum_{\text{St}}W_{\text{st}}\times {\bar{y}}_{\text{st}}$$

**Table 1:** Summary of sampling and video analysis

Area	Depth (m)	# Sites	Area (km^2^)^a^	# Living *Ostrea edulis*	Dead *Ostrea edulis*
Area 1	0.5–3	22	15.93	1,011	541
	3–6	16	6.37	38	121
	6–10	15	8.68	6	29
Area 2	0.5–3	24	13.35	423	383
	3–6	33	6.42	728	773
	6–10	20	10.42	18	104
Area 3	0.5–3	32	12.35	162	368
	3–6	38	6.78	28	107
	6–10		8.93		
Area 4	0.5–3	26	20.65	54	54
	3–6	34	7.91	14	131
	6–10		12.91		
Koster area	0.5–3	59	7.47	435	1,301
	3–6	55	5.86	337	1,067
	6–10	61	7.93	4	101
Total area	All	435	138.90	3,258	5,080

Note

The distribution of sampled sites, area size, and counted number of living and dead *Ostrea edulis* individuals for each area and depth stratum.

a The areas of 0.5–3 m strata were corrected by subtracting 1/6 from the area of 0–3 m.

and.

(2) $$V\left({\bar{y}}_{\text{total}}\right)=\sum_{\text{St}}\frac{W_{\text{st}}^{2}\times s_{\text{st}}^{2}}{n_{\text{st}}}$$

where $W_{\text{st}}$, $s_{\text{st}}^{2}$, and $n_{\text{st}}$ is the weight (areal proportion), the variability, and number of samples of a stratum. Finally, the population total (as opposed to the mean) and its associated error are calculated as:

(3) $${\bar{y}}_{\text{total}}\times N\pm \sqrt{V\left({\bar{y}}_{\text{total}}\right)}\times N$$

where *N* is the total extent of the area investigated with representative samples (Cochran, 1977).

## 3 RESULTS

### 3.1 General observations and description of habitats

**Figure 3:**
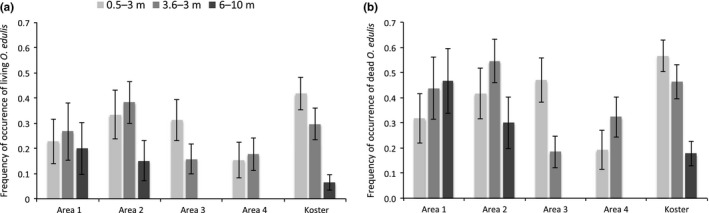
Frequency of occurrence of (a) living and (b) dead *Ostrea edulis* (mean ± *SE*)

The field survey resulted in data from a total of 435 sites: 260 in the coastal area and 175 in the Koster area (Table 1). In total, we identified and counted 3,258 living and 5,080 dead *O. edulis*. Overall, living *O. edulis* was widely distributed and present in all areas and depth strata (Figure 3a). Highest frequencies of living oysters were observed in the shallowest stratum where oysters occurred in 25% of the sites, followed by 19% of the intermediate depth sites and 6% of the 6–10 m sites in the whole study area. The corresponding values for dead *O. edulis* were generally higher: 36%, 32%, and 19%, respectively (Figure 3b).

**Figure 4:**
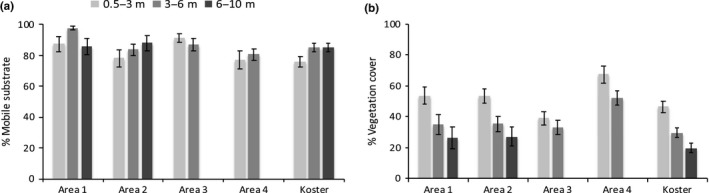
Distribution and coverage of (a) mobile substrate and (b) vegetation across the five areas and three depth intervals used in the study (mean ± *SE*)

Substrate composition showed substantial variability among sites and some differences among areas and depth strata (Figure 4a). Within the depth intervals and levels of exposure relevant to this study, the bottom consisted mainly of mobile substrates (81%, 85%, and 86% in each of the three depth strata). Overall, 60%–75% of the substrates were characterized as mud or sand and 10% and 5% were gravel and shell hash, respectively. In shallow habitats, vegetation covered on average 50%–60%, while 30%–50% and 20%–40% cover was observed in the two deeper strata (Figure 4b). The most frequently occurring species of vegetation at sampling sites were *Chorda filum* and *Zostera marina,* which both occur in shallow soft sediment habitats (28 and 25%, respectively). *Fucus serratus*, *Halidrys siliquosa*, and *Saccharina latissima* were the most common species in rocky substrates (22%, 21%, and 13% of all transects). All coverage ratios presented in this section correspond to mean values for the whole study area.

**Figure 5:**
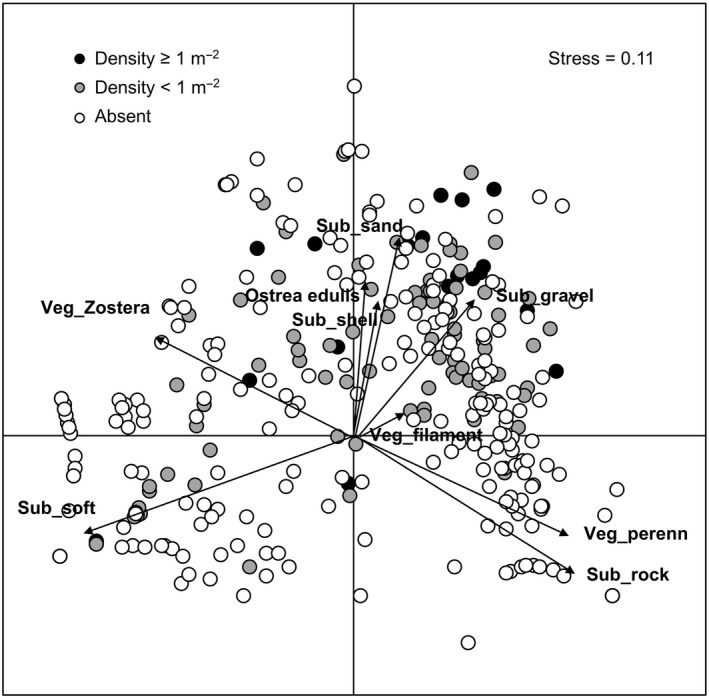
Nonmetric MDS of benthic habitats (vegetation = Veg_x and substrate = Sub_x) using data from video‐analyses of 435 sites along the Swedish west coast

Multivariate analyses of abundances of *O. edulis*, vegetation and substrate cover as revealed from the video‐analyses, show that the occurrence of oysters, and particularly high abundances, is associated with large cover of sand, shell hash, and gravel (Figure 5). There is also an overlap between low abundances of *O. edulis* and the occurrence of *Z. marina* in soft sediments. In contrast, oysters were not found in habitats with a dominance of rocky substrates with perennial algae, nor in habitats dominated by unvegetated soft sediments. In summary, the overall pattern that emerges is that *O. edulis* is a common and sometimes abundant species in areas of intermediate exposure dominated by unvegetated, sandy, and gravelly substrates on the Swedish west coast.

### 3.2 Abundance and population estimates

**Figure 6:**
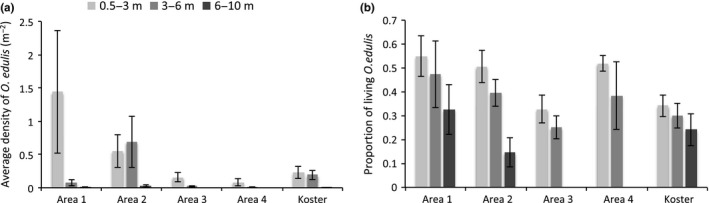
The geographical and vertical distribution of *Ostrea edulis* in the study area illustrated by variation in (a) average density and (b) the ratio of living/(living + dead) *Ostrea edulis* (mean ± *SE*)

**Figure 7:**
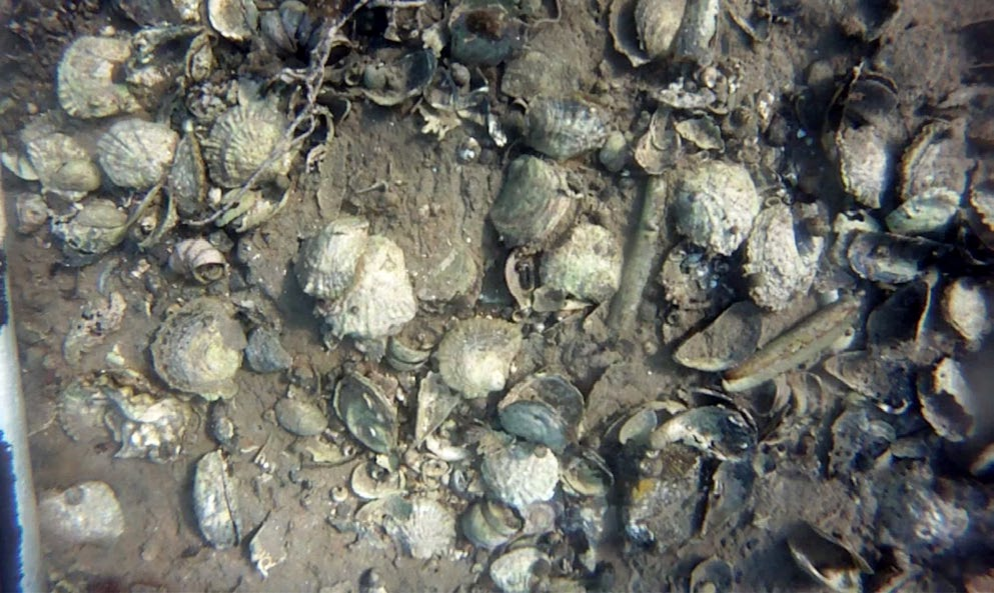
A still frame from the site with highest average density of *Ostrea edulis*. One of the sled runners appears to the left of the picture

Overall analyses of *O. edulis* abundance revealed significant variability among geographical areas and among depths (Table 2). The largest mean abundances of *O. edulis* were found in the two northern coastal areas (i.e., Area 1 and Area 2; Figure 6a). The highest average density of living *O. edulis* in a single given video transect was measured to 31.6 living *O. edulis*/m^2^ (Figure 7). The corresponding value for dead oysters was 15 m^−2^. The sampling method used limited the possibility of estimating the proportion of oysters that formed clumps, but there was still no doubt that the majority of the oysters in the video transects were solitary. At high‐density sites, clumps of up to six living and dead oysters were, however, observed.

**Table 2:** Analyses of variance and a posteriori tests (Student–Neuman–Keuls) of abundance of *Ostrea edulis* and proportion of living *O. edulis* (only in sites containing oysters)

Living *O. edulis*						Ratio Living/(Living + Dead) *O. edulis*				
	*df*	MS	*F*	*p*		*df*	MS		*F*	*p*
Area = A	4	3.86	2.73	.03		4	0.68		0.74	.56
Depth = D	2	18.13	12.73	.00		2	6.76		7.45	.00
A × D	6	0.46	0.33	.92		6	0.70		0.77	.60
Site (A × D)	424	1.41	8.79	.00		156	0.91		4.51	.00
Residual	433	0.16				106	0.20			
	*O. edulis*								Ratio	
SNK of area	Area 4 = Area 3 = Koster = Area 1 = Area 2					SNK of depth			D3 < D2 = D1	
SNK of depth	D3 = D2 < D1									

Note

Data were transformed as ln(*X* + 1) in order to conform to homogeneity of variances.

The ANOVA detected significant differences between areas, but the SNK was not powerful enough to logically resolve differences among individual areas (Table 2). In contrast, comparisons among depths showed that there were significantly higher abundances at depths 0.5–3 m compared to the deeper strata. Apart from variability due to fixed factors, there was also substantial and significant variability among sites within regions and depths.

Analysis of the spatial patterns of the proportion living *O. edulis* indicated that the proportion of living oysters is significantly smaller at 6–10 m (Table 2). Inspection of means showed consistent tendency for larger proportions of dead *O. edulis* with increasing depths in all areas (Figure 6b). Thus, oysters appear to survive better at shallower depths or are transported by waves or currents to deeper areas following their death.

The size of the *O. edulis* population in Skagerrak was estimated to 36.6 ± 16.3 million individuals (Total population ± *SE*; Table 3). Assuming an average weight of 63 g per individual (Sallén Lennerthson & Lindegarth, unpublished data), the biomass was roughly estimated to 2.3 ± 1.0 thousand tonnes in the region. Almost 80% of the population was found between 0.5 and 3 m, while only 2% is found below 6 m (Table 3). Overall, the standard error of the mean estimate is approximately 50% of the mean. The high uncertainty of this estimate is a reflection the large natural, partly unpredictable variability in abundance among sites, prevalent in this species.

**Table 3:** Estimated population size of *Ostrea edulis* in different areas and depth strata based on estimates of abundance and areal extent using Equations (1), (2), (3)

Area	0.5–3 m	3–6 m	6–10 m	Total	*SE*
Koster	1.69	1.18	0.02	2.89	1.00
Area 1–4	27.54	5.34	0.79	33.67	16.26
Total	29.23	6.52	0.81	**36.56**	16.29

Note

Total population in the study area marked in bold.

**Figure 8:**
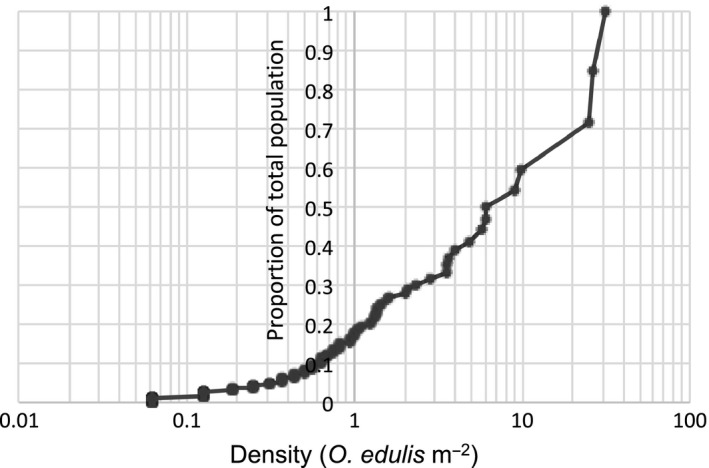
The cumulative proportion of the *Ostrea edulis* population as a function of density

One aspect of the large spatial variability among sites is that a large proportion of the population is concentrated to a few particularly important sites. Thus, we examined the relative importance of high‐density and low‐density sites in the area by plotting the cumulative proportion of the population ordered from low‐ to high‐density transects against the abundance (Figure 8). This analysis showed that approximately 60% of the *O. edulis* population is found at sites with five or more individuals/m^2^, that is, in densities defined as oyster bed according to (OSPAR, 2009). In this particular dataset, this corresponds to eight out of 159 transects having living *O. edulis* (~5%) (Figure 8) or < 1% of all sampled sites.

## 4 DISCUSSION

Sustainable management of the European flat oyster *O. edulis* requires information on distribution, status, and potential threats, both in a national Swedish context and throughout its indigenous range (OSPAR, 2009). We used a nondestructive sampling method and a stratified random sampling approach to quantify abundance and distribution of the native *O. edulis* population throughout its main distribution area in Sweden. These data have been absent, and as a consequence, the population has often been overlooked in comprehensive assessments (e.g., OSPAR, 2009). However, we found that the species can be considered frequent throughout the region and the population size indicates that it contributes significantly to the strongly decimated North Sea population of *O. edulis* (Airoldi & Beck, 2007; OSPAR, 2009). These results have a number of implications for the conservation and management of *O. edulis* populations in general, and the management of the local fishery in particular. But we also believe that the study can be used to illustrate important general challenges for the management of marine benthic habitats, especially when monitoring habitats and species without much previous knowledge, and therefore be a key to sustainable management of valuable marine resources.

Despite the fact that *O. edulis* populations still exist throughout its original biogeographic range, population sizes and abundances are considered severely decimated and fragmented. The estimated population size of 36.6 ± 16.3 million individuals, abundances of up to 30 oysters/m^2^, clumps of up to 6 living and dead individuals, and a frequency of ≈40% in some parts of the area suggest that the status of Swedish population is relatively good compared to most populations in Europe. Additionally, the status may also have improved in a historical perspective, as a low genetic variability in the population indicates some degree of inbreeding following population bottlenecks or, at least, lower population numbers in the past (Johannesson et al., 1989). Although abundances are highly variable, they are within the range of better‐studied *O. edulis* populations in the North Sea, for example, those in the Limfjord region in Denmark, and in Ireland (Kristensen & Hoffmann, 2006; Smyth et al., 2009; Tully & Clarke, 2012). The highly heterogeneous distribution of the Swedish population, where rare (<1% of the sampled sites) oyster beds constitutes 60% of the population, resembles the Irish more than the geographically closer Limfjord population. In Ireland, (Tully & Clarke, 2012) estimated that 80% of national population was found in one 4‐km^2^ site, Inner Tralee, while the Limfjord population is characterized by large low‐density areas (Kristensen & Hoffmann, 2006).

Previous knowledge about the environmental requirements of *O. edulis* in Sweden is largely qualitative and anecdotal (but see Bodvin et al., 2011). This study shows that high densities of oysters occur mainly in sandy–gravelly sediments at depths shallower than 3 m, but never directly on rocks or boulders. Interestingly, this seems to differ from preferences previously reported for this species in the North Sea region, for example, in Ireland and Northern Ireland (Barry, 1981; Kennedy & Roberts, 1999). This lack of solid quantitative information about the species environmental requirements and settlement preferences in combination with the absence of comprehensive geographic information on substrates and vegetation had direct consequences for the design of the sampling program. In order to achieve a complete assessment of population size including estimates of uncertainty, the sampling design had to be representative and randomized with respect to all environmental factors except depth strata (distribution and area calculated from digital nautical charts). This is in contrast to most recent surveys of *O. edulis* populations, which have been based on present or historical oyster beds (Kristensen & Hoffmann, 2006; UMBS, 2007; Tully & Clarke, 2012). Such studies result in cost‐efficient and accurate estimates of the population in these beds, but provide no information on oysters outside these selected areas. Other population size assessments have been based on the distribution of suitable *O. edulis* bottom substrate throughout the study area (Kennedy & Roberts, 1999; Smyth et al., 2009). In order to provide reliable estimates, such approaches assume solid knowledge about the species’ habitat requirements and of the distribution of these conditions throughout the targeted area.

While previous knowledge did not allow for any a priori stratification based on habitat suitability in this study, data on *O. edulis* occurrence and substrate characteristics were collected and used to assess the robustness of the completely randomized approach. Thus, assuming that a suitable oyster habitat was characterized by (a) the presence of living or dead *O. edulis* or (b) mobile substrates, we applied the method used by Kennedy and Roberts (1999) to estimate total population size (Table S1–S4). These scenarios resulted in population estimates of 40.4 and 40.7 million, respectively, which is very close to that obtained without any assumptions of habitat suitability (36.6 ± 16.3 million oysters). Therefore, we conclude that the method not only appears robust but also allowed for straightforward estimation of error.

Another methodological feature of this study was the comprehensive use of video to assess more than 400 sites on the Swedish Skagerrak coast. This choice was based on the need for a nondestructive and cost‐efficient sampling method, due to a large sampling area with a high proportion of protection area (Marine National Park or Natura 2000). With few exceptions (Grizzle, Brodeur, Abeels, & Greene, 2008), subtidal oyster populations are otherwise sampled using either habitat destructive dredging (Tarnowski, 2005; Greco, 2009) or by more expensive scuba diving (Kennedy & Roberts, 1999; Soniat et al., 2014). As an initial assessment of a previously poorly studied population of *O. edulis*, we believe that towed video was a useful approach. And despite being a nutrient‐rich coastal area, turbidity was not considered an aggravation factor, probably due to the short distance between the camera and the seafloor (Rein, Schoeman, Brown, Quinn, & Breen, 2012). The method was, however, not without limitations. For example, first‐year oysters (<3–4 cm) are generally too small to be identified (Sallén Lennerthson and Lindegarth, unpublished data) and consequently these cannot be assessed accurately. Based on the findings of this study, high‐density areas or beds could, however, be selected and studied in more detail (i.e., demographic structure and spatfall), using, for example, scuba diving. Furthermore, previous evaluations of the method have shown that burial and coverage under other oysters or vegetation may lead to underestimation of abundance by on average 20% (Thorngren et al., 2017). Using common practice for corrections of sampling efficiency (Powell, Ashton‐Alcox, & Kraeuter, 2007; Tully & Clarke, 2012) that would mean the estimated size of the Swedish *O. edulis* population may be raised by a factor of 1.25–45.7 million adult oysters. Note, however, that the dominating source of uncertainty is the prevalence of spatial variability, which is on the order of 45%.

One major purpose of estimating the size of the Swedish *O. edulis* population was to use it as tool for assessing the sustainability of current levels of exploitation and management practice in general. The Swedish food agency reports that on average 88,000 *O. edulis* were landed annually in the last 5 years which, for a population of 36.6 million oysters, is equivalent to an annual catch of 0.24%. Even in the unlikely event that the illegal and private fishing would be of the same magnitude as the licensed catch, the level of exploitation would be ~ 0.5% annually. As a comparison, the total allowable catch (TAC) in the MSC certified *O. edulis* fishery in the Limfjord, Denmark, was set to 450 tonnes for a biomass of 2,648 tonnes (i.e., 17% of the stock) in 2011 (Andrews, Maar, & Brand, 2017). Although demographics are not entirely accounted for, it appears that on a regional scale, the current level of exploitation in Sweden is sustainable in relation to the population size. Importantly, this conclusion extends to the fishing methods used, as fishing in this area is done manually by diving and the use of dredges or trawls has long been forbidden. This measure has contributed to a reduced risk for habitat degradation in the area, which has been identified as one of the major threats to oyster populations (OSPAR, 2009).

Considering the relative health of the Swedish population compared to most populations in Europe, the present management efforts of oysters in Sweden are surprisingly limited. For example, the catches are not regulated by quotas and although a large part of these coastal areas benefit from various types of protective status, such as being included in the Natura 2000 network, these are generally not defined as no‐take zones with respect to *O. edulis* (except for Kosterhavet National Park). Nevertheless, apart from the dredging ban, two additional important aspects of Swedish oyster management deserve to be mentioned. First, the vast majority of the potential *O. edulis* habitats in Sweden have been privately owned for at least three centuries and oysters can therefore not be collected without permission from the landowner (:19). This may have contributed to restraining exploitation pressure over the years and provides additional support to the notion that resource ownership can create strong incentives for a sustainable fishing pressure by avoiding the “tragedy of the commons” (Hardin, 1968). Even though there are differences in terms of number of landowners and the level of active management, similar conclusions were reached regarding Scotland's only remaining oyster production area in Loch Ryan. This population has also been privately owned for centuries, and the landlord in Loch Ryan has used an active approach to management, for example, through seeding, harrowing, and monitoring and by issuing catch limits (Eagling, Ashton, & Eagle, 2015). In contrast, none of these measures have been taken by the many landowners in Sweden. Second, the prevention, control, and surveillance of infectious diseases are well developed. Since 1995 the National Veterinary Institute has continuously examined the presence of *B. ostreae* and *Marteilia refringens* on the Swedish west coast, strict rules for translocation and import of bivalves have been implemented and there has also been a duty to report bonamiosis (SJVFS, 1995:49). These measures can potentially have contributed to the absence of these parasites in Swedish waters (Jordbruksverket, 2010), which is of high importance for the viability of both natural oyster beds and aquaculture, as these parasites have caused massive mortality outbreaks in Europe (Abollo et al., 2008).

Although the status of the population at present appears to be good, numerous challenges must be addressed to ensure the future persistence and sustainable management of *O. edulis* in Sweden. These include safeguarding existing populations by defining protected areas and maintaining fishing mortality at sustainable levels, preventing the spread of infectious diseases and pathogens. We also need to monitor the dispersal and population development of *M. gigas* and further investigate the interaction between the two oyster species in this region. This is particularly relevant in this area, as the habitats in which you only find *O. edulis*, according to a survey in 2018, are declining rapidly (personal obs.), and because the conditions in these specific habitats (subtidal and horizontal) can provide a competitive advantage for *M. gigas* (Zwerschke et al., 2018). In addition, this initial assessment of the *O. edulis* population should be followed by more detailed monitoring of the denser oyster beds, for example, in terms of demographic structure and recruitment. We have presented the first comprehensive study of *O. edulis* in the Swedish Skagerrak region. Due to its representative design and quantitative approach, it has a great potential to serve as a benchmark for knowledge‐based, adaptive management in a future with many existing and emerging threats.

## CONFLICT OF INTEREST

None declared.

## AUTHOR CONTRIBUTIONS

LT, TDH, and ML conceived the ideas and designed methodology; LT and TDH collected the data; LT, TDH, PB, and ML analyzed the data; LT and ML led the writing of the manuscript, but PB and TDH contributed with comments and manuscript improvements. All authors contributed critically to the drafts and gave final approval for publication.

### OPEN DATA BADGE

This article has earned an https://openscience.com for making publicly available the digitally‐shareable data necessary to reproduce the reported results. The data is available at https://osf.io/jgpxw/?view_only=d070b45802a4426da028efffde3d0f76.

## Data Availability

All video analysis data are available through the Open Science Framework https://osf.io/jgpxw/?view_only=d070b45802a4426da028efffde3d0f76
