# Supplementary material for: Assessment of the population of Ostrea edulis in Sweden: A marginal population of significance?
Source: Ecol Evol. 2019 Nov 19;9(24):13877–88. doi: 10.1002/ece3.5824 (PMC6953678; doi:10.1002/ece3.5824)
Supplement: Supplementary file 1 [file ECE3-9-13877-s001.pdf]

Supporting information

An estimation of the total *Ostrea edulis* population in Sweden, based on assumptions about suitable habitats adopted (but slightly modified) from Kennedy and Roberts (1999), where the following two scenarios were assessed: (1) suitable habitats is assumed to be all mobile substrates or (2) suitable habitat is characterised by presence of living or dead *O. edulis*. The total number of oysters is calculated as:

$$P = \sum_{i=1}^{h} (R_i \times F_i \times \overline{D}_i).$$

where P is the total population of *O. edulis* in the study area and h is the number of spatial units (i.e. combinations of different geographical areas and depth strata). R_i is the estimated areal extent of each unit in m^2 (Table S1), F_i is the proportion of suitable habitat in each unit (Table S2) and $\overline{D}_i$ is the mean oyster density in each subarea in individuals m^{-2} (Table S3). Resulting population sizes under the two scenarios are shown in Table S4.

Table S1. Areal extent of spatial units. R_i ($m^2 \times 10^6$):

Depth (m)	Area 1	Area 2	Area 3	Area 4	Koster
0.5–3	15.24	13.30	12.35	14.16	7.43
3–6	6.35	6.41	6.20	6.60	5.86
6–10	8.65	10.45			7.93

Table S2. Proportion of suitable substratum present in the subarea, F_i.
a) Scenario 1

Depth (m)	Area 1	Area 2	Area 3	Area 4	Koster
0.5–3	0.87	0.78	0.91	0.77	0.76
3–6	0.98	0.83	0.87	0.80	0.85
6–10	0.86	0.88	NA	NA	0.85

b) Scenario 2					
0.5–3	0.36	0.42	0.47	0.19	0.58
3–6	0.50	0.58	0.18	0.35	0.47
6–10	0.47	0.30	NA	NA	0.18

Table S3. Mean density of *O. edulis* per spatial unit (m^{-2}), $\bar{D}_i$.

a) Scenario 1

Depth (m)	Area 1	Area 2	Area 3	Area 4	Koster
0.5–3	1.65	0.71	0.17	0.11	0.30
3–6	0.08	0.83	0.03	0.02	0.23
6–10	0.01	0.03	NA	NA	0.00

b) Scenario 2					
0.5–3	3.95	1.32	0.34	0.34	0.39
3–6	0.15	1.20	0.13	0.04	0.41
6–10	0.03	0.09	NA	NA	0.01

Table S4. Number of *O. edulis* in each subarea (million individuals).

a) Scenario 1

Depth (m)	Area 1	Area 2	Area 3	Area 4	Koster	Total
0.5–3	21.9	7.3	2.0	1.2	1.7	34.1
3–6	0.5	4.4	0.1	0.1	1.1	6.2
6–10	0.1	0.3	NA	NA	0.0	0.4
Total	22.5	12	2.1	1.3	2.8	40.7

b) Scenario 2						
0.5–3	21.9	7.3	2.0	1.2	1.7	33.8
3–6	0.5	4.4	0.1	0.1	1.1	6.2
6–10	0.1	0.3	NA	NA	0.0	0.4
Total	22.5	12	2.1	1	2.8	40.4
